# Respiratory metabolites in bronchoalveolar lavage fluid (BALF) and exhaled breath condensate (EBC) can differentiate horses affected by severe equine asthma from healthy horses

**DOI:** 10.1186/s12917-020-02446-9

**Published:** 2020-07-08

**Authors:** Marilena Bazzano, Luca Laghi, Chenglin Zhu, Gian Enrico Magi, Beniamino Tesei, Fulvio Laus

**Affiliations:** 1 School of Biosciences and Veterinary Medicine, University of Camerino, Via Circonvallazione 93/95, 62024 Matelica, MC Italy; 2 Department of Agro-Food Science and Technology, Centre of Foodomics, University of Bologna, Bologna, Italy

**Keywords:** Metabolomics, Asthma, Bronchoalveolar lavage fluid, Exhaled breath condensate, Horse

## Abstract

**Background:**

The use of an untargeted metabolomic approach to investigate biofluids of respiratory origin is of increasing interest in human and veterinary lung research. Considering the high incidence of equine asthma (> 14%) within horse population and the importance of this animal model for human disease, we aimed to investigate the metabolomic profile of bronchoalveolar lavage fluid (BALF) and exhaled breath condensate (EBC) in healthy and asthmatic horses.

**Results:**

On the basis of clinical, endoscopic and BALF cytology findings, 6 horses with severe asthma (Group A) and 6 healthy horses (Group C) were included in the study. ^1^H-NMR analysis was used to identified metabolites in BALF and EBC samples. Metabolomic analysis allowed to identify and quantify 12 metabolites in BALF and seven metabolites in EBC. Among respiratory metabolites, myo-inositol, formate, glycerol and isopropanol in BALF, and methanol and ethanol in EBC, differed between groups (*p* < 0.05).

**Conclusions:**

The application of metabolomic studies to investigate equine asthma using minimally invasive diagnostic methods, such as EBC metabolomics, provided promising results. According to our research, the study of selective profiles of BALF and EBC metabolites might be useful for identifying molecules like myo-inositol and methanol as possible biomarkers for airways diseases in horses.

## Background

Respiratory metabolomics is gaining popularity in human and pediatric medicine [1, 2]. Metabolic dysfunctions in chronic lung diseases such as asthma, chronic obstructive pulmonary disease, idiopathic pulmonary fibrosis and cystic fibrosis have been ascertained [2–6], and clinicians are now using metabolomics in the diagnostic and prognostic approach other than in clinical research [2]. This new approach also shows a great potential in veterinary medicine [7–9], and untargeted metabolomics is often applied to improve knowledge and speculate novel hypothesis in human and veterinary lung research [10, 11].

About 14% of adult/old horses naturally develop an asthma-like disease known as equine asthma [12]. Human and equine asthma share several common features like recurrent airway obstruction, bronchial hyperresponsiveness and airways inflammation [13]. Despite the fact that the respiratory cytological reaction is predominantly eosinophilic in men and neutrophilic in horses, equine asthma is a recognized model for human disease [14, 15]. As in humans, the cause of equine asthma remains not completely understood, being the result of a broad range of immunological, inflammatory and biochemical perturbations [16–18]. In people, metabolomics differentiates between asthmatic and healthy subjects, and different metabolomic endotypes of asthma have been recognized [18]. In horses showing respiratory symptoms compatible with asthma, the diagnosis is currently based on the presence of abnormal bronchoalveolar lavage fluid (BALF) cytology [19], but molecular biomarkers of lung inflammation would offer new insight for pathogenetic and diagnostic advances. The collection of exhaled breath condensate (EBC) has recently emerged as a non-invasive sampling method to obtain information about the health status of the respiratory system in humans and equines [11, 20, 21]. Previous studies investigated biomarkers of oxidative stress [21], hydrogen peroxide content and pH variations in EBC of horses [20], and only one study performed the metabolomic analysis of equine EBC [11].

The metabolomic profile of exhaled breath condensate (EBC) and tracheal wash (TW) in horses with asthma has been previously investigated [11]. In the current study we aimed to evaluate the respiratory metabolites in BALF and EBC of healthy horses and horses affected by severe equine asthma (sEA). Our goal was to obtain data that would improve the current knowledge on the potential of metabolomic analysis and biomarkers for the diagnosis and treatment of equine airways diseases.

## Results

Endoscopic evaluation of airways during BALF collection revealed the presence of tracheal mucus (mean score > 2) [22] and thickening of tracheal septum in horses from group A, whereas normal airways aspect was found in control group. According to BALF cytology, mean neutrophils percentage was 47.1% (±22% standard deviation) in horses with sEA and 9.4% (±0.8% standard deviation) in control group, respectively [23].

**Fig. 1:**
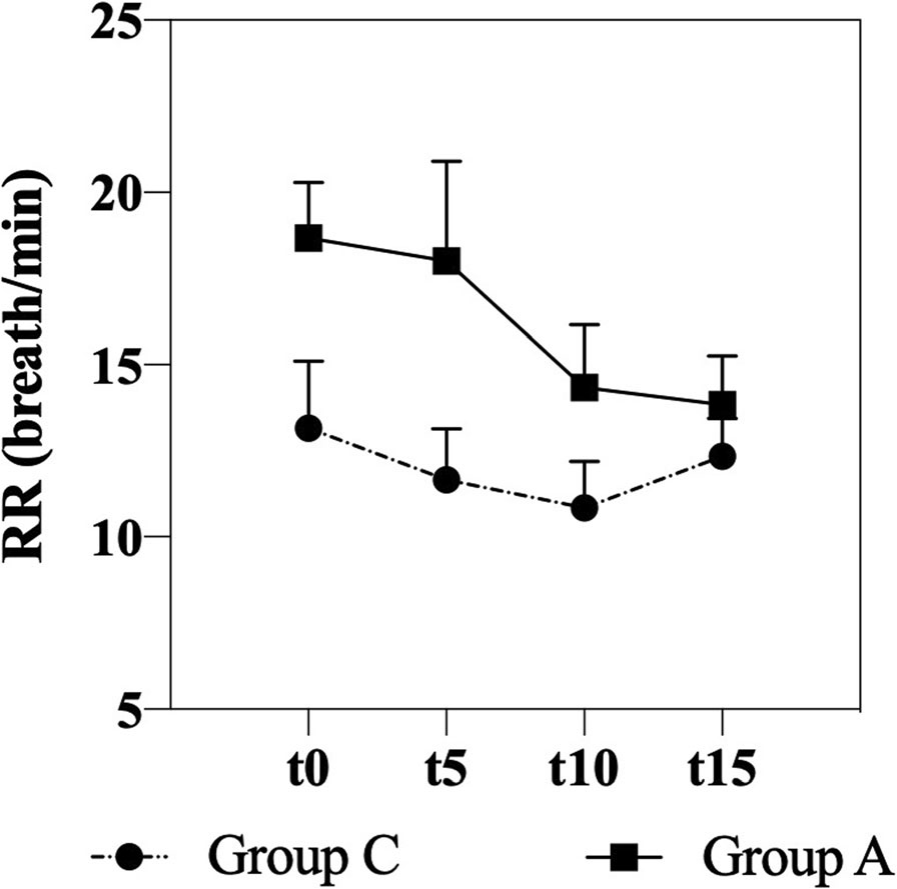
Mean values and SEM of respiratory rate (RR) recorded every 5 minutes during EBC collection in control (Group C) and asthmatic horses (Group A)

**Fig. 2:**
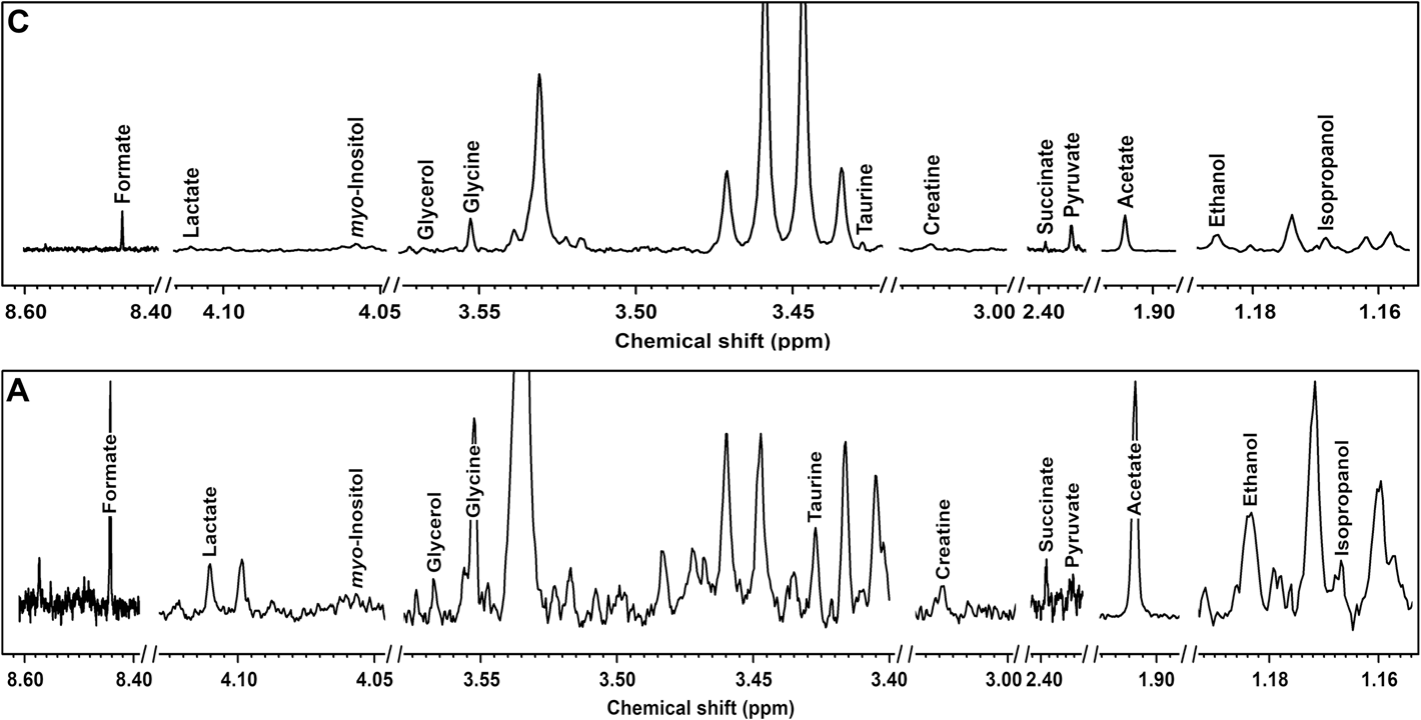
Examples of the spectra obtained by ^1^H-NMR analysis from BALF of asthmatic horses **a** and controls **c**

**Fig. 3:**
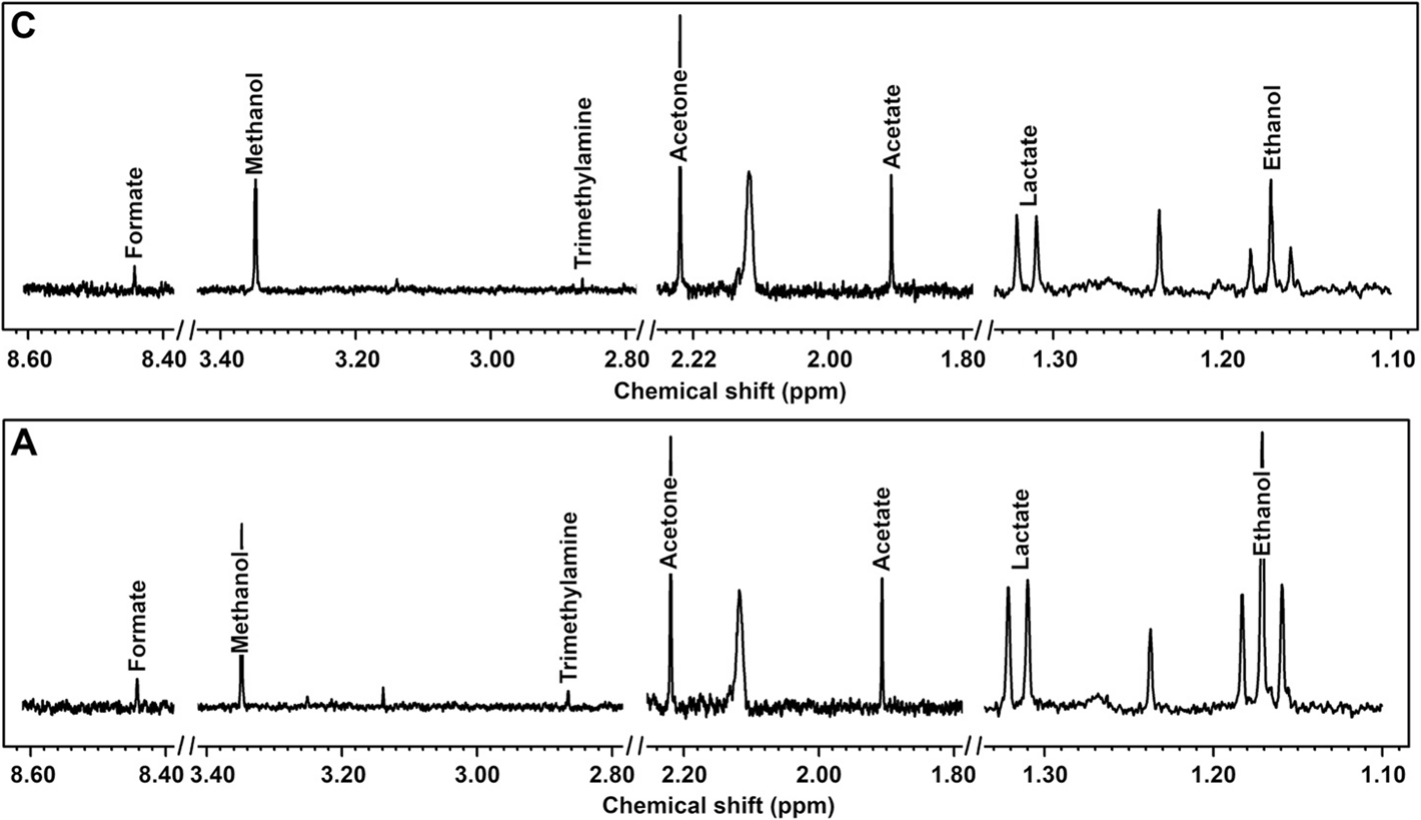
Examples of the spectra obtained by ^1^H-NMR analysis from EBC of asthmatic horses **a** and controls **c**

Respiratory rate (RR) and pattern remained unchanged at rest and during EBC collection in both groups. The respiratory pattern clinically observed was a normal breathing (eupnea) in healthy subjects, and a thoracoabdominal asynchrony in sEA horses (respiratory effort). Despite a significant difference in RR between Group C and Group A (*p* = 0.029), no significant change was observed during EBC collection within each group (Fig. 1). The median volumes of EBC collected over 15 min ranged from 1.1 ml in Group C to 1.750 ml in Group A. ^1^H-NMR identified 12 metabolites in BALF (Table 1): formate, lactate, myo-inositol, glycerol, glycine, taurine, creatine, succinate, pyruvate, acetate, ethanol and isopropanol (Fig. 2). Metabolites found in EBC (Table 2) included methanol, ethanol, formate, trimethylamine, acetone, acetate and lactate (Fig. 3).

**Table 1:** Metabolites’ concentrations (mmol/L), expressed as mean ± standard deviation, quantified by ^1^H-NMR in bronchoalveolar fluid (BALF) samples of control group (Group C) and asthma group (Group A). 95% CI for each group is indicated in brackets. *P* values are indicated for each metabolite, statistical significances (*) are in bold letters

Metabolites	Group C (***n*** = 6)	Group A (***n*** = 6)	***P*** values
Formate*	1.17E-02 ± 1.77E-03 (1.02E-02 – 1.31E-02)	1.21E-02 ± 5.01E-03 (8.05E-03 – 1.61E-02)	**0.040**
Lactate	1.42E-02 ± 3.88E-03 (1.11E-02 – 1.73E-02)	1.29E-02 ± 2.12E-03 (1.12E-02 – 1.46E-02)	0.210
Myo-inositol*	2.82E-02 ± 1.70E-02 (1.46E-02 – 4.18E-02)	1.58E-02 ± 5.88E-03 (1.11E-02 – 2.05E-02)	**0.036**
Glycerol*	1.55E-03 ± 1.55E-03 (3.13E-04 – 2.80E-03)	1.03E-03 ± 3.12E-04 (7.78E-04 – 1.28E-03)	**0.003**
Glycine	5.48E-03 ± 1.43E-03 (4.33E-03 – 6.62E-03)	4.46E-03 ± 1.44E-03 (3.31E-03 – 5.61E-03)	0.994
Taurine	6.32E-03 ± 4.44E-03 (2.77E-03 – 9.88E-03)	1.03E-02 ± 9.45E-03 (1.78E-03–2.73E-02)	0.123
Creatine	1.60E-03 ± 8.37E-04 (2.27E-04–9.3E-03)	1.55E-03 ± 6.60E-04 (1.02E-03 – 2.08E-03)	0.615
Succinate	8.89E-04 ± 1.29E-04 (7.86E-04 – 9.92E-04)	8.49E-04 ± 2.26E-04 (1.03E-04–6.69E-03)	0.245
Pyruvate	9.46E-04 ± 2.96E-04 (7.09E-04 – 1.18E-03)	7.57E-04 ± 2.70E-04 (5.41E-04 – 9.74E-04)	0.845
Acetate	1.42E-02 ± 1.22E-03 (1.33E-02 – 1.52E-02)	1.41E-02 ± 2.18E-03 (1.23E-02 – 1.58E-02)	0.230
Ethanol	4.60E-03 ± 1.28E-03 (3.58E-03 – 5.62E-03)	4.61E-03 ± 7.68E-04 (3.99E-03 – 5.22E-03)	0.289
Isopropanol*	1.53E-03 ± 3.16E-04 (1.28E-03 – 1.79E-03)	3.66E-03 ± 5.22E-03 (5.18E-04 – 7.84E-03)	**< 0.001**

**Table 2:** Metabolites’ concentrations (mmol/L), expressed as mean ± standard deviation, quantified by ^1^H-NMR in exhaled breath condensate (EBC) samples of control group (Group C) and asthma group (Group A). 95% CI for each group is indicated in brackets. *P* values are indicated for each metabolite, statistical significances (*) are in bold letters

Metabolites	Group C (***n*** = 6)	Group A (***n*** = 6)	***P*** values
Formate	2.38E-03 ± 4.63E-04 (2.01E-03 - 2.75E-03)	2.30E-03 ± 6.56E-04 (1.77E-03 - 2.82E-03)	0.463
Methanol*	1.13E-02 ± 3.07E-03 (8.87E-03 - 1.38E-02)	1.84E-02 ± 8.39E-03 (1.17E-02 - 2.51E-02)	**0.046**
Trimethylamine	1.46E-04 ± 7.13E-05 (8.90E-05 - 2.03E-04)	1.78E-04 ± 1.35E-04 (6.97E-05 - 2.85E-04)	0.189
Acetone	2.72E-03 ± 1.27E-03 (1.70E-03 - 3.74E-03)	2.99E-03 ± 9.31E-04 (2.25E-03 - 3.74E-03)	0.510
Acetate	3.38E-03 ± 7.02E-04 (2.82E-03 - 3.94E-03)	2.79E-03 ± 6.01E-04 (2.31E-03 - 3.27E-03)	0.742
Lactate	9.50E-03 ± 8.40E-03 (2.78E-03 - 1.62E-02)	5.34E-03 ± 2.48E-03 (3.35E-03 - 7.33E-03)	**0.018**
Ethanol*	1.31E-02 ± 9.32E-03 (5.69E-03 - 2.06E-02)	3.60E-02 ± 5.68E-02 (0–8.15E-02)	**0.001**

**Fig. 4:**
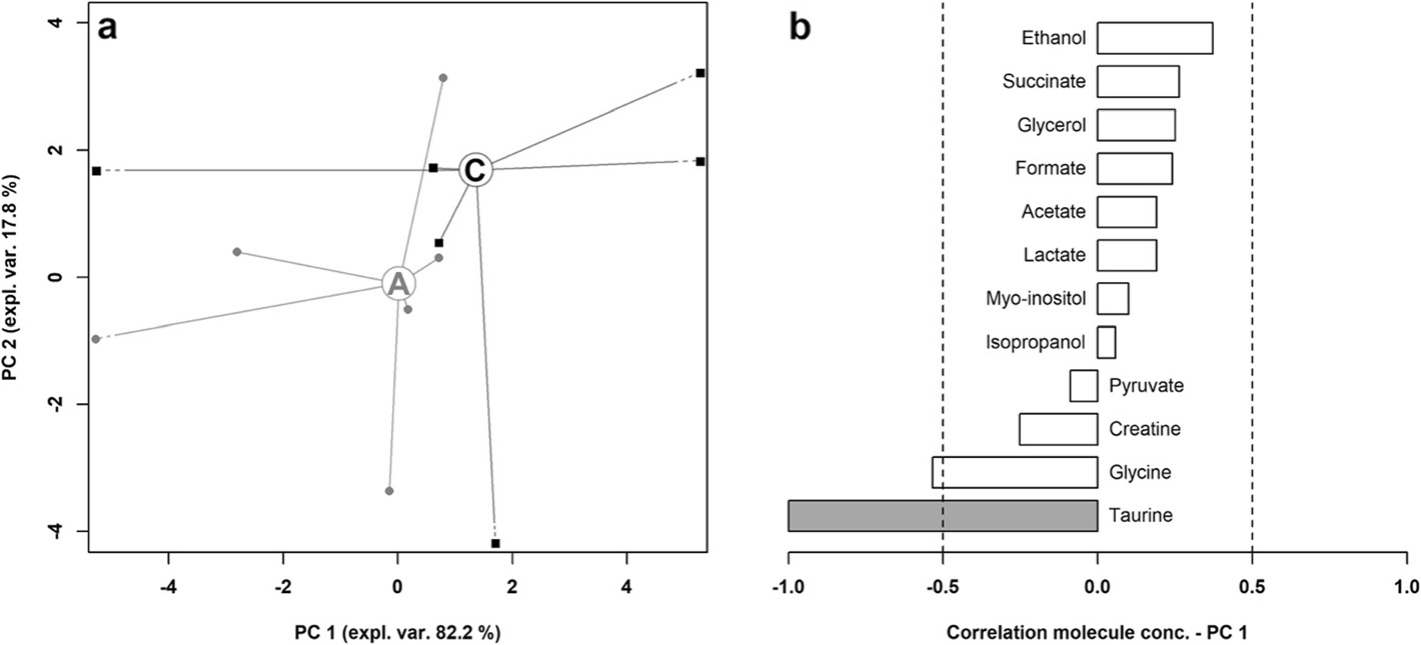
Robust Principal component analysis of the BALF metabolome: **a** Scoreplot of an rPCA model calculated on the space constituted by the concentration of each molecule quantified in BALF samples. Empty circles highlight the median values for asthma **a** and control **c** groups **b** Bar plot describing the correlation between the concentration of each molecule and its importance along PC 1.

**Fig. 5:**
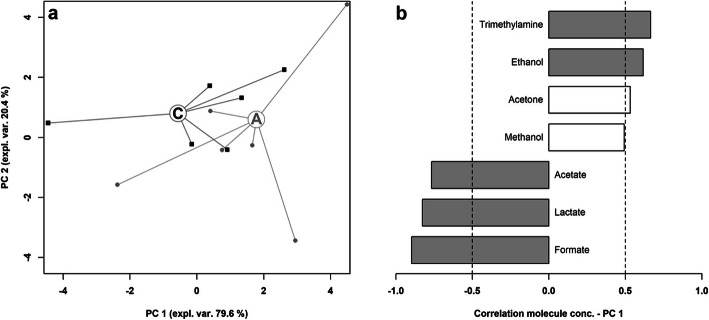
Robust Principal component analysis of the EBC metabolome: **a** Scoreplot of an rPCA model calculated on the space constituted by the concentration of each molecule quantified in EBC samples. Empty circles highlight the median values for asthma **a** and control **c** groups. **b** Bar plot describing the correlation between the concentration of each molecule and its importance along PC 1

Overviews of the main features of both metabolites’ profiles can be obtained by robust principal component analysis. In the case of BALF (Fig. 4), the first principal component (PC 1), summarizes the main differences between asthmatic and control horses, characterized by lower and higher PC 1 scores, respectively. The corresponding correlation plot shows that the molecules mainly responsible for such grouping are taurine and, to a lower extent, glycine. Focusing of EBC (Fig. 5), PC 1 is the component mainly accounting for the differences between the samples from asthmatic and control horses. Asthmatic horses appear at higher PC 1 scores, mainly because of the high concentration of trimethylamine and acetone and of the low concentrations of formate, lactate and acetate.

## Discussions

The current study contributes to improve the knowledge on the metabolomic profile of equine respiratory system [11] by providing useful information about BALF and EBC metabolomics in healthy horses and horses with severe asthma.

Despite horses included in the control group were found to have an average of neutrophils in BALF just below the limits that allows to exclude the presence of inflammation, the absence of clinical signs, anamnesis and endoscopic findings related to respiratory diseases made up for the slight individual variability in neutrophil percentage that could be accounted to environmental factors [23].

Among metabolites found in BALF samples, four molecules differed significantly between healthy horses and horses affected by asthma. Group A had significant lower levels of myo-inositol, a metabolite that promotes maturation of pulmonary surfactant and supports respiratory function [24]. Inositol modulates cytoskeleton dynamics thus promoting a mechanical stabilization of cell shape and allowing alveolar cells to counteract collapsing forces [25]. In people, myo-inositol recruits organic compound and water in the alveolar space for the formation of a biofilm layer at the interface, thereby decreasing surface tension [26]. Previous research in infants demonstrated that a dramatic decrease in plasma levels of myo-inositol is associated to a higher risk of respiratory distress syndrome [27]. Its use at pharmacological doses provided promising preliminary results in humans [24] and has been recently proposed for a phase III clinical trial [28]. Myo-inositol is also the most effective allosteric effector identified to date, being able to increase the tissue delivering of oxygen bound to hemoglobin [29]. Since its administration can improve sport performance in laboratory mice, its analogues have been suspected to be abused in horse racing industry [30] but no study about its effect in equine species has been performed. In our opinion, further research about myo-inositol function and its efficacy as therapeutic tool in horses with respiratory diseases should be performed.

Glycerol was found in BALF of patients affected by acute respiratory distress syndrome (ARDS) in a recent study [31] and has also been detected (but not quantified) by ^1^NMR in lung of healthy humans, pigs, mice and rats [32]. The role of glycerol in respiratory diseases is not clear, however it could be linked to the alteration of lipid metabolism observed in BALF samples from asthmatic and ARDS-affected patients [2, 33–35].

Higher formate concentrations have been found in BALF of asthmatic horses compared to healthy subjects. Formate is the only non-tetrahydrofolate-linked intermediate in one-carbon metabolism [36] produced in mammals from a variety of metabolic sources, in different tissues and from different substrates. Formate plays a critical role in the three canonical functions of one-carbon metabolism: purine nucleotide synthesis, thymidylate synthesis, and the provision of methyl groups for synthetic, regulatory, and epigenetic methylation reactions [36]. Formate also plays a significant role in embryonic growth and high formate concentrations were found in fetal lambs indicating a role in fetal development [36]. Deficiency in either folate or vitamin B12 significantly increases formate levels [37] explaining why vegetarian people have higher formate levels than non-vegetarians [38]. A single previous study detected formate by NMR in BALF of ARDS-affected patients, but no control group was used in that report [31]. Since there was no reason to suspect vitamin deficiency in the animals included in our study, the role of formate and its variation in respiratory system of healthy and asthma affected horses need further investigation to be clarified. In our opinion this kind of evaluation should also be referred to different species, since there seem to be considerable species differences in synthesis/disposal mechanism of formate [36].

A significant increase in isopropanol was observed in horses with asthma compared to healthy subjects. A metabolomic study performed by NMR found increased isopropanol levels in the lungs of naphthalene-exposed mice potentially due to the loss of cell membrane integrity in the airway epithelial cells of mice [39].

Although not statistically different between groups, we found metabolites in BALF samples including lactate, pyruvate, taurine and glycine that have been reported to have a role in the pathogenesis of allergies. Taurine and glycine, the main molecules determining the spreading of BALF data according to the rPCA model, have a protective effect and regulate cytokine over-expression in allergies [2]. Lactate and pyruvate are representative of energy metabolism that was found to be altered in BALF from mice with experimentally induced allergic airway inflammation [34]. Also the presence of creatine might suggest the promotion of energy metabolism via the urea cycle, and substantial increase of creatine in lungs have been observed following sepsis and silica exposure [34]. Although these metabolites have been found also in equine BALF, further research is necessary to better elucidate their role in equine respiratory system.

The possibility to collect biological samples from respiratory origin in standing non sedated horses without coercion and in a totally noninvasive way represented the major advantage of using EBC to investigate respiratory disease in horses. Notwithstanding this considerable advantage, the lack of standardized collection methods represents a major limitation in the use of this specimen for diagnostic purpose in clinical practice. Furthermore, the absence of commercially available devices for EBC collection, the influence of environmental and animal factors, and the paucity of studies about equine EBC having high variability in sampling procedures (time, temperature, type of condensation chamber) should be taken into account when interpreting EBC results.

According to our findings, the use of metabolomic analysis on EBC samples provided evidence of a different metabolomic profile in horses with sEA compared to healthy subjects. The higher methanol concentrations found in Group A indicate an active inflammatory status of airways of horses affected by asthma [11, 40] and substantially confirmed our findings in a previous study on EBC metabolomics [11].

The significant increase in ethanol concentration in Group A might also be related to the presence of pulmonary disease. This metabolite was found to be higher in exhaled breath of patients suffering from pulmonary cystic fibrosis compared to healthy subjects [41].

Studies on EBC metabolomics reported increased acetate and acetone levels in patients suffering from pulmonary disease [42], in our study we observed slightly higher, though not statistically significant, concentrations of acetone in Group A whereas no difference was observed between healthy and asthmatic horses in acetate levels.

## Conclusions

The discovery of biomarkers for the diagnosis and treatment of respiratory diseases and the need for additional asthma metabolomic studies to explore these issues using minimally invasive diagnostic methods, such as EBC metabolomics, are key topics in respiratory research both in human and veterinary medicine [19, 42, 43]. Although the limited number of subjects enrolled in the study represented a main limitation and did not consent to investigate the potential influence of gender on BALF and EBC metabolome, our data suggest that selective profiles of BALF and EBC metabolites might be useful for identifying molecules like myo-inositol and methanol as possible biomarkers for equine airways diseases. According to the present and previous studies [11], metabolomic analysis has a great potential to better explain the pathophysiology of equine asthma, but further studies are necessary to investigate the role of these metabolites allowing researchers to adopt novel diagnostics and therapeutic strategies to treat the disease.

## Methods

### Animals

On the basis of clinical presentation, endoscopic findings (mucus score > 2) [44] and BALF cytology (neutrophils > 25%) [45], 6 horses affected by sEA (2 geldings, 4 mares, 15 ± 4 years, 423 ± 66 Kg), selected among patients referred to the Veterinary Teaching Hospital of Camerino University (Italy), were included in asthma group (Group A). Typical respiratory symptoms [45, 46] were present at clinical examination (e.g. exercise intolerance, crackles and wheezes, increased respiratory effort) and all horses have been shown respiratory signs for at least 1 month before the beginning of the study. No drugs had been administered in the previous 2 months.

Six healthy horses (3 geldings, 2 mares, 1 male, 17 ± 5 years, 445 ± 46 Kg) with no history of respiratory symptoms in the last 3 years were included in control group (Group C).

All horses were housed indoors in individual shavings bedded boxes at the Veterinary Teaching Hospital of Camerino for at least 1 week before the beginning of the study.

Animals were fed 8 ± 1 kg hay/day and water was provided ad libitum.

The study was conducted during September–October 2018. A written consent was obtained from all horse owners before the study began. After the end of the experimental period all the horses returned to their stables. All experimental procedures were approved by the Animal Care Committee of Camerino University (Registration number: E81AC.8.B, March 1st, 2018) and were in accordance with the standards recommended by the EU Directive 2010/63/EU for experiments on animals.

### BALF and EBC samples collection

BALF samples were collected as described previously [23]. Briefly, after horse sedation with detomidine (10 μg/Kg BW) and butorphanol (10 μg/Kg BW), a 220 cm long endoscope, with outer diameter of 1.2 cm (Mercury Endoscopia Italiana) was passed through the ventral meatus of the nasal cavity, larynx and carina, and edged in a left segmental bronchus (Additional file 1). Always the same two operators, one at the control and one at the insertion section, handled the scope. Pre-warmed sterile saline solution (250 ml) was instilled through the work channel of the scope and immediately retrieved (at least 50% of the instilled volume). One BALF aliquot (200 μl) was used for cytologic evaluation as previously described [47]. According to cytological appearance of BALF cytospin specimens, a grading of airways inflammation based on neutrophils percentage was performed. A second BALF aliquot (2 ml) was cooled at 5 °C and stored at − 80 °C, within 30 min from collection, and analyzed by ^1^H-NMR within 2 weeks. After each BALF sampling, the endoscope was cleaned with 3% hydrogen peroxide solution, followed by 10% iodopovidone solution, afterwards it was rinsed with saline solution and, lastly, with sterile distilled water.

**Fig. 6:**
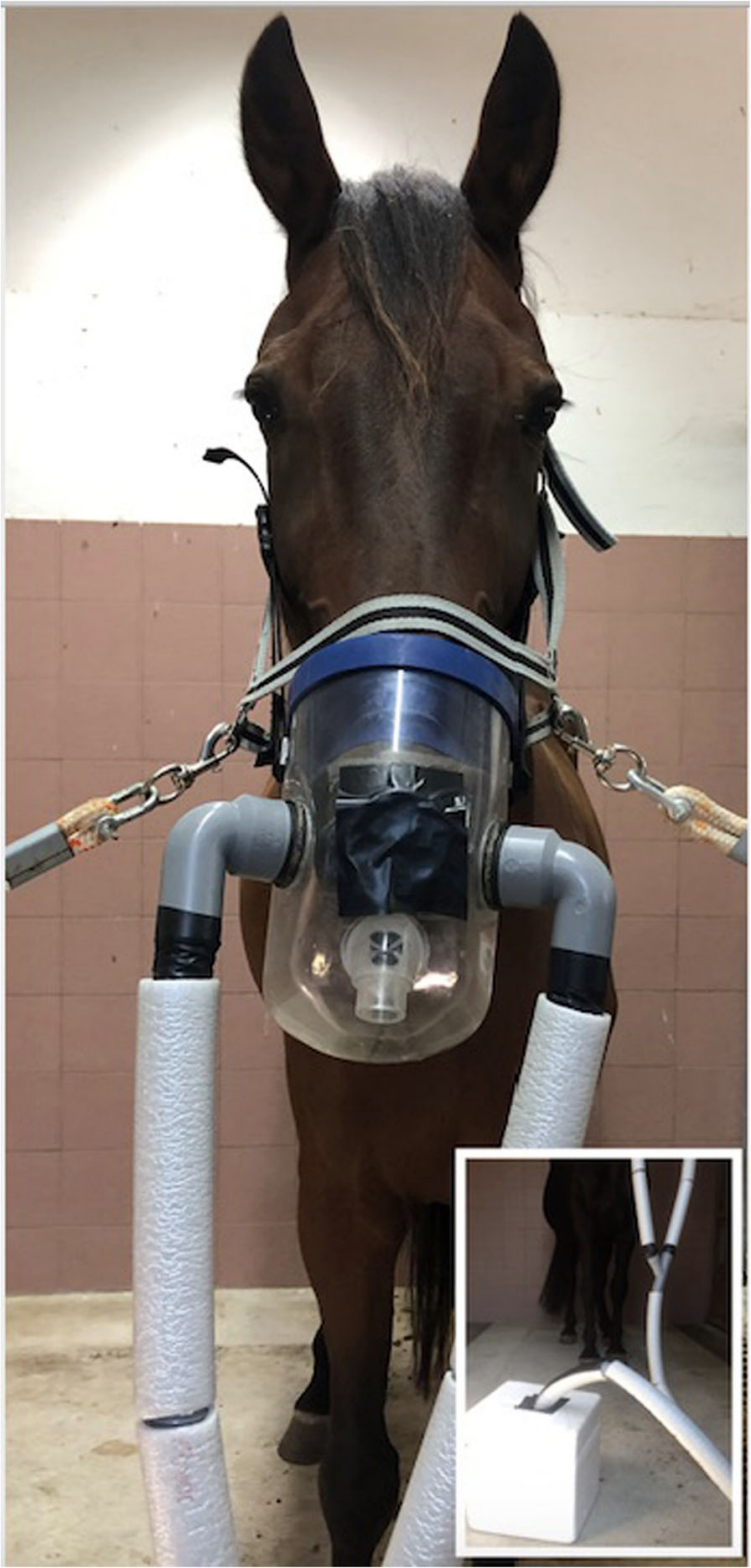
EBC collection performed using a condensation system consisting of a modified aerosol face mask connected via tubing to a condensation chamber

EBC samples were collected using a condensation system consisting of an aerosol face mask connected via tubing to a condensation chamber as previously described [11]. Briefly, the modified aerosol face mask (SM Trade&Technology SRL) had three unidirectional valves, one valve allowing the air to enter during inspiration and other two valves connected via thermally insulated tubing to a condensation device that allowed expired air to unidirectionally pass through the system. The flexible plastic tubes (length: 280 cm; radius: 2.1 cm) were coated with thermal insulating tubes to maintain the temperature of expired air, thus preventing air condensation inside the tubing system. The condensation chamber consisted of a 500 mL glass becker inserted into an ice block, positioned inside of a thermally insulated box, having a one-way valve on the top to prevent EBC contamination by retrograde flow of environmental air (Fig. 6). During EBC collection the temperature inside the condensation chamber was monitored by means of a suitable thermometer (− 20 °C). Before starting EBC sampling, all animals were accustomed to the face mask that was well accepted by the horses.

Every EBC collection was performed indoors (12 ± 2 °C), before feeding, between 9.00–09.30 am, after an accurate cleaning of face and nostrils, without any sedation and before BALF collection (Additional file 2).

EBC samples were collected over a 15 min period, immediately cooled at 5 °C and stored at − 80 °C, within 30 min from collection, and analyzed by ^1^H-NMR within 2 weeks.

Respiratory rate and respiratory pattern were monitored continuously throughout the EBC collection. After each EBC collection, the mask was cleaned with water and single use absorbent paper towels at first, thereafter mask, tubes and becker were rinsed with deionized water, wiped with single use paper towel and then left air drying.

### Metabolome analysis by ^1^H-NMR

Each BALF and EBC sample was prepared for NMR analysis by thawing, followed by centrifugation at 18640 g and 4 °C for 15 min [11]. A 0.7 mL aliquot of supernatant was added to 0.1 mL of a D_2_O solution of 3-(trimethylsilyl)-propionic-2,2,3,3-d4 acid sodium salt (TSP) 10 mM, buffered at pH 7.00 ± 0.02 by means of 1 M phosphate buffer. The D_2_O solution contained also NaN_3_ 2 mM, to avoid microorganisms’ proliferation. Afterwards, each sample was centrifuged again at the above conditions.

^1^H-NMR spectra were recorded at 298 K with an AVANCE III spectrometer (Bruker, Milan, Italy), at a frequency of 600.13 MHz. A CPMG-filter, set as suggested by Bazzano et al. [11] allowed reducing the signals from large molecules, while the residual water signal was suppressed by presaturation. Each spectrum was acquired by summing up 256 transients registering 32 K data points over a 7184 Hz spectral window, with acquisition time of 2.28 s and relaxation delay of 5 s. The ^1^H-NMR spectra were adjusted for phase and baseline distortions as described by Foschi et al. [48].

The signals were assigned by comparing their chemical shift and multiplicity with Chenomx software library (Chenomx Inc., Canada, ver 8.3). Quantification of each molecule was achieved by rectangular integration, by focusing on one signal per molecule free from superimpositions. The added TSP, at a known concentration, was employed as internal standard in the first sample analyzed. Differences in water content among samples were then taken into consideration by probabilistic quotient normalization (PQN) [49]. In detail, each spectrum has been normalized towards the reference one through PQN in two steps. First, for each molecule the change of concentration with respect to the reference has been calculated, in order to obtain a distribution of changes. Second, the median value of this distribution has been considered as the unspecific change caused by water, and therefore removed.

### Statistical analysis

Statistical analysis was performed by using PRISM 8 (GraphPad Software Inc.) and R statistical software (www.r-project.org).

Two-way repeated measures ANOVA was performed to highlight difference in respiratory rate between Group A and C during EBC collection.

After Box Cox data normalization [50], two-sided F-test was applied to assess statistical differences in metabolites’ concentrations between asthma and control groups and 95% confidence interval (CI) was calculated [51]. Statistical significance was established at *p <* 0.05.

The trends underlying the metabolome of BALF and EBC were summarized by means of robust principal component analysis (rPCA) [52]. This algorithm rotates the original space represented by molecules’ concentrations, to show the samples from the point of view representing the greatest portion of the samples’ variance. The scoreplot, the representation of the samples from the new point of view, helps to inspect the trends underlying the samples, while the loadingplot shows the Pearson’s correlation between the concentration of each molecule and its importance along the principal components, thus evidencing the molecules mainly driving the trends. For the purpose, a significance at *p* < 0.05 was accepted.

## Supplementary information

**Additional file 1.** BALF collection.mov: This video shows the collection of BALF in a horse included in the study.

**Additional file 2.** EBC collection.mov: This video shows the collection of EBC in a horse included in the study.

## Data Availability

The datasets analysed during the current study are available from the corresponding author on reasonable request.

## Abbreviations

BALF: Bronchoalveolar lavage fluid
EBC: Exhaled breath condensate
1HNMR: Proton nuclear magnetic resonance
TW: Tracheal wash
SD: Standard deviation
RR: Respiratory rate
ARDS: Acute respiratory distress syndrome
TSP: Acid sodium salt
PQN: Probabilistic quotient normalization
CI: Confidence interval
rPCA: Robust principal component analysis
